# Mobile Apps for COVID-19: A Systematic Review of Reviews

**DOI:** 10.3390/healthcare12020139

**Published:** 2024-01-08

**Authors:** Felix Holl, Johannes Schobel, Walter J. Swoboda

**Affiliations:** DigiHealth Institute, Neu-Ulm University of Applied Sciences, 89231 Neu-Ulm, Germany; johannes.schobel@hnu.de (J.S.); walter.swoboda@hnu.de (W.J.S.)

**Keywords:** SARS-CoV-2, mobile applications, mHealth, telemedicine, systematic review

## Abstract

Background: One measure national governments took to react to the acute respiratory syndrome coronavirus type 2 (SARS-CoV-2) pandemic was mobile applications (apps). This study aims to provide a high-level overview of published reviews of mobile apps used in association with coronavirus disease 19 (COVID-19), examine factors that contributed to the success of these apps, and provide data for further research into this topic. Methods: We conducted a systematic review of reviews (also referred to as an umbrella review) and searched two databases, Medline and Embase, for peer-reviewed reviews of COVID-19 mobile apps that were written in English and published between January 1st 2020 and April 25th 2022. Results: Out of the initial 17,611 studies, 24 studies were eligible for the analysis. Publication dates ranged from May 2020 to January 2022. In total, 54% (*n* = 13) of the studies were published in 2021, and 33% (*n* = 8) were published in 2020. Most reviews included in our review of reviews analyzed apps from the USA, the UK, and India. Apps from most of the African and Middle and South American countries were not analyzed in the reviews included in our study. Categorization resulted in four clusters (app overview, privacy and security, MARS rating, and miscellaneous). Conclusions: Our study provides a high-level overview of 24 reviews of apps for COVID-19, identifies factors that contributed to the success of these apps, and identifies a gap in the current literature. The study provides data for further analyses and further research.

## 1. Introduction

The first reports about a novel coronavirus in Wuhan, China, were published in February 2020 [1,2]. The pathogen, which is now called severe acute respiratory syndrome coronavirus type 2 (SARS-CoV-2), caused severe respiratory symptoms, including fever, dizziness, and cough. The World Health Organization (WHO) declared SARS-CoV-2 a global pandemic on the 11th of March 2020 after the virus spread internationally and the number of cases outside of China increased 13-fold within two weeks [3]. Almost three years later, a dashboard presented by Dong et al. [4] registers over 676,000,000 infections and 6,881,955 deaths globally as of March 10th 2023 [5]. As growing parts of the population were ill with coronavirus disease 19 (COVID-19), clinical capacities were endangered, requiring national governments to act. Especially before vaccines were widely available, government-imposed measures ranged from basic hygiene recommendations, such as the mandatory wearing of medical masks, to lockdowns that interrupted life for up to several weeks [6,7].

The use of mobile applications in health, also known as mHealth, has gained significant attention in recent years. mHealth has been shown to have a positive impact on chronic disease management, including cardiovascular diseases and diabetes mellitus [8]. It offers advantages such as convenience, potential cost-effectiveness, and scalability, making it an attractive option for patient communication, monitoring, and education [9]. Additionally, mHealth tools have been observed to impact patients who are less inclined to engage with traditional health services, thus improving access to healthcare [9]. However, the use of mHealth in improving health outcomes faces barriers, particularly in developing countries, including technical, financial, political, social, ethical, and cultural challenges [10]. Despite these barriers, mHealth has shown potential in infectious disease control. They are seen as a valuable tool for the early detection and monitoring of infectious diseases [11,12,13]. Therefore, mHealth presents a promising avenue for enhancing healthcare delivery, particularly in the context of chronic disease management and infectious disease control.

Apps have already proven themselves effective in tracking and containing the viral spread in previous endemic situations, such as with Ebola [14], malaria, and tuberculosis [11]. Governments started to develop mobile applications (apps) for COVID-19 to support their efforts in containing the pandemic, such as information sharing, symptom monitoring, and contact tracing [15,16,17]. In parallel, researchers analyzed these apps to assess them either in general or regarding their suitability for the purpose they were developed for. Consequently, a lot of reviews have been performed to synthesize the results of these studies [18,19,20,21,22]. However, these reviews usually focus on a narrow aspect of apps for COVID-19 and to our knowledge, no work has yet synthesized the findings of all the reviews in the form of a systematic review of reviews, also referred to as an umbrella review [23].

### Objective

The primary objective is to identify reviews on the subject of mobile apps for COVID-19 to provide a high-level overview of what has been published on the topic since the beginning of the pandemic. The aim is to provide an overview of the different public review articles about apps for COVID-19 for the academic community. We seek to identify gaps in the evidence on a metalevel, investigate factors that contributed to the success of the apps, and provide recommendations for possible future epidemic situations. The data collected through this review can serve as a basis for future studies to further investigate the gaps and success factors and address new research questions.

## 2. Materials and Methods

### 2.1. Systematic Literature Search

We conducted a systematic review of reviews structured according to the Preferred Reporting Items for Systematic Reviews and Meta-Analyses (PRISMA) statement from 2020 [24]. To organize the review process and reduce possible bias, we created a review protocol according to the PRISMA-P extension for review protocols [25,26], which is presented in the Supplementary Materials Table S1.

The population, intervention, control, outcome (PICO) [27] framework was used to define inclusion and exclusion criteria. As shown in Table 1, we searched for reviews of COVID-19-related apps designed for people who had possibly been exposed to SARS-CoV-2 and/or people diagnosed with COVID-19. We included any kind of review articles, which included both reviews of the academic literature and apps. Reviews provide a comprehensive and critical evaluation of existing research, aiming to summarize, analyze, and synthesize the current state of knowledge on a specific topic. Studies that were not reviews, such as any kind of primary research, e.g., surveys, were excluded. The identified reviews were then qualitatively analyzed using their metadata, subject, used methods, and results. Reviews were included with or without any comparator.

To further qualify for inclusion, publications needed to be reviews, without specifying any kind of review, available in the English language, peer-reviewed, and published between January 1st 2020 and April 25th 2022, the start date to attribute to the onset of the pandemic. We searched the two databases, Medline and Embase, using fixed search strings that were created using features identified through PICO (see Table 2). The search strings were developed by one researcher (M.A.) and validated by another researcher (F.H.). We consulted a librarian from Neu-Ulm University of Applied Sciences (T.G.) during the development of the search string. The strings were also externally validated by two other researchers (M.F. and J.K.) who have previously published systematic reviews. Medline was accessed through the PubMed interface [28]. We did not conduct any complementary searches. We searched two general literature databases after consulting an information specialist and optimized the yield and minimized the effort of searching for evidence for this umbrella review according to the recommendations by Golder and Wright [29].

Our search strategy followed five steps: 1. database search; 2. title/abstract screening; 3. retrieval of eligible publications; 4. full-text screening; and 5. analysis. For step 1, no filters or limitations were used. During steps 2 and 4, the two reviewers (F.H. and M.A.) were unaware of each other’s decisions, and conflicts were then discussed until consensus was achieved. If consensus had not been reached through the discussion, a third reviewer (J.S.) would have reviewed the paper and made a final decision. In step 4, the reason for exclusion was documented. The reasons for exclusion were as follows: wrong intervention (*n* = 22), wrong type of paper (*n* = 20), wrong language (*n* = 2), and wording outcomes and wrong setting (each *n* = 1). A list of reports excluded during full-text screening, along with reasons for exclusion, is available in the Supplementary Materials Table S3.

### 2.2. Data Extraction

Our search results were exported in text or .ris file formats. *EndNote20* was used for literature management. Entries were imported into the systematic review software *Covidence* [30] for screening and data extraction.

The data extraction and quality assessment templates were created in *Covidence* [30]. The template includes a study’s metadata, subject, methods, and results. As an initial means of categorization, we recorded whether studies included literature reviews, app reviews, or both. Our template for quality assessment consists of items from the A Measurement Tool to Assess Systematic Reviews (AMSTAR) [31]. We used version 2 of AMSTAR, which was created for the assessment of randomized clinical studies [31] and includes sixteen items in total. As this review does not contain clinical studies, the items of AMSTAR were removed because they did not fit the context. Both extraction and quality assessment templates are available in Supplementary Materials Tables S4 and S5.

### 2.3. Quality Assessment

The quality of included reviews was assessed using the AMSTAR 2 tool, a validated instrument for appraising the quality of systematic reviews [31]. AMSTAR2 covers 16 domains, of which 7 are considered critical. Of the remaining 9 items, 3 are considered critical.

Critical domains are considered especially influential for review validity. The remaining critical domains are as follows: (1) protocol pre-registration (item 2), (2) literature search strategy (item 4), and (3) list and justification for excluded studies (item 7). Each included review was rated for adequacy on each domain as either “Yes”, “No”, or “Partial Yes” (available only for domains 2, 4, 7, and 8).

The fulfillment of each dimension across the different reviews was assessed using a table. Based on these domains, we also assigned a summary quality rating as “critically low” (≥2 “no” ratings on the critical domain), “low” (≤1 “no” ratings on critical domains), “moderate” (≥2 “no” ratings on non-critical domains), or “high” (≤1 “no” on a non-critical domains) to each review.

### 2.4. Data Synthesis and Analysis

One researcher (M.A.) manually extracted the data and another researcher (F.H.) validated the extractions. Conflicts were discussed until consensus was achieved. After data extraction, studies were assigned keywords that described their topic and methodology. We then grouped studies based on the similarity of these keywords.

Some reviews applied the Mobile Application Rating Scale (MARS) by Stoyanov et al. [32], which is a widely used questionnaire-based tool that assesses app quality [33,34]. Overall app quality is further divided into four dimensions “Engagement”, “Functionality”, “Information quality” and “Aesthetics”. To compare the results of studies using MARS, we analyzed the MARS total scores of all included apps and mean scores per dimension.

## 3. Results

### 3.1. Systematic Literature Search

**Figure 1:**
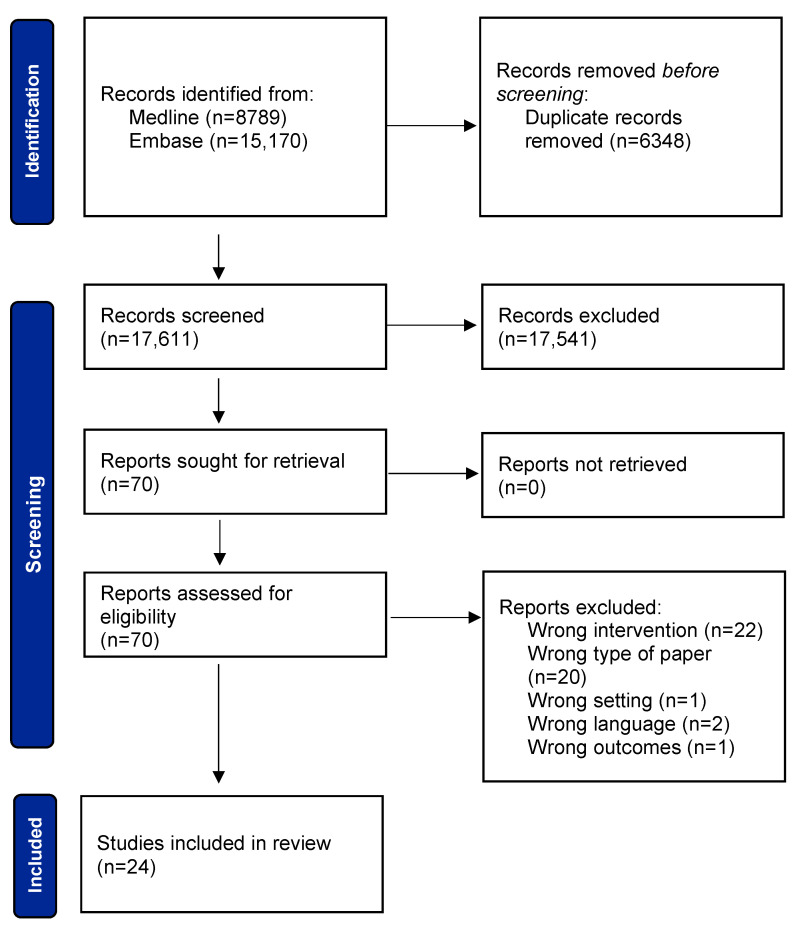
The PRISMA flowchart illustrates step by step how we identified the 24 studies to include in our analysis.

A total of 23,959 records were found via database searches. Figure 1 shows the number of records resulting from each search step. Medline was searched on April 23th 2022 and Embase on April 25th 2022. After the automated removal of duplicates via *Covidence* [30], 17,611 records were manually screened according to title and abstract, resulting in 70 remaining reports. After full-text screening and the exclusion of another 46 reports, the 24 studies from Table 3 were included in the analysis.

### 3.2. Spatiotemporal Analysis

**Figure 2:**
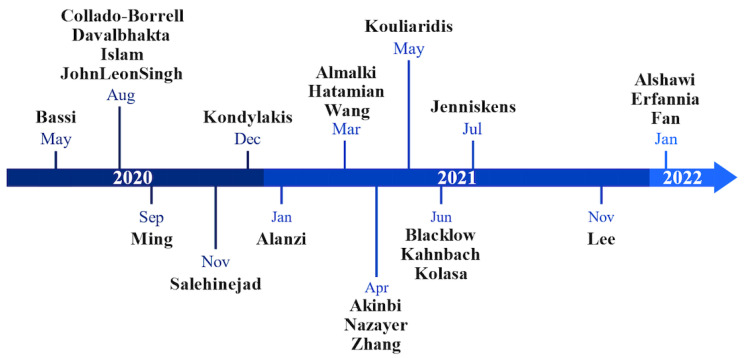
The timeline of published reviews on apps for COVID-19 included in our review.

Figure 2 illustrates a timeline of the 24 articles included in our review by publication date. The publication dates range from May 2020 to January 2022. In total, 54.2% (*n* = 13) of the studies were published in 2021; 33.3% (*n* = 8) of the studies were published in 2020. The other three studies were published in 2022 (*n* = 3, 12.5%). The timeline demonstrates how most reviews were published between May 2020 and July 2021, with visibly fewer publications after July 2021.

**Figure 3:**
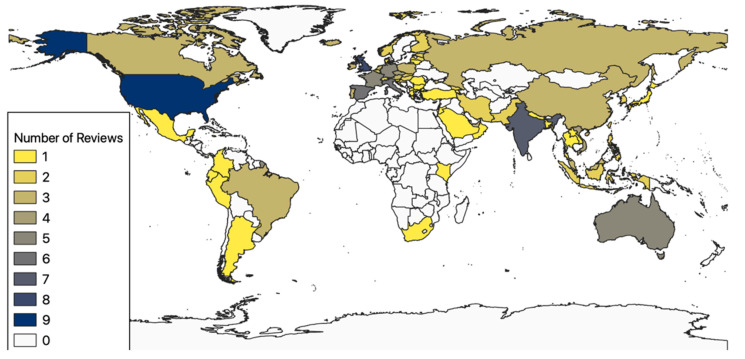
Visualization of the number of reviews in which apps from the respective countries were examined.

Figure 3 visualizes a geographical analysis of the number of reviews in which apps from the respective countries were examined.

The map shows that most reviews were on apps from the United States (*n* = 9), the United Kingdom (*n* = 8), and India (*n* = 7). Among other origin countries of the analyzed apps are Australia (*n* = 5), Singapore (*n* = 4), and Vietnam (*n* = 4), as well as Canada, Brazil, China, Malaysia, Russia, and the United Arab Emirates (*n* = 3 each). There were also many European countries (including the United Kingdom, Italy (*n* = 6), Spain (*n* = 6), and Germany and France (*n* = 5 each)). Few reviews analyzed apps from African countries or Central America. Several reviews analyzed apps from more than one country.

### 3.3. Categorization

**Figure 4:**
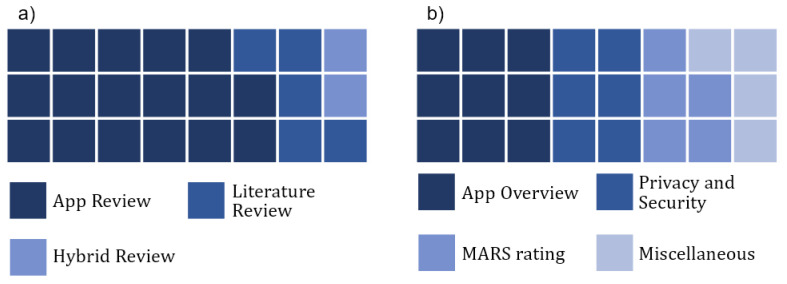
(**a**) Publications were either reviews of apps, literature reviews, or both: (**b**) categorization resulted in four categories.

Figure 4a shows how most publications (*n* = 17; 71%) were reviews of apps followed by literature reviews (*n* = 5; 21%) and hybrid reviews (*n* = 2; 8%). We defined hybrid reviews as reviews that include both the literature and apps. We grouped the included reviews into four categories (visualized in Figure 4b). These categories include reviews that provide an overview of published apps (*n* = 9; 37.5%), focus on privacy and security (*n* = 6; 25%), and use MARS for rating apps (*n* = 5; 21%) and those that did not fit into any of the previous categories (*n* = 4; 16.5%).

In the following sections, which are structured according to our categorization, we narratively describe the key outcomes of the included studies. We provide a table with all the extracted data in the Supplementary Materials.

#### 3.3.1. App Overview

The largest category was “app overview” (*n* = 9). Included reviews [18,20,21,35,36,37,38,39,40] share the primary purpose of identifying available apps for COVID-19 and summarizing their features. In total, 55% (*n* = 5) of these reviews [18,21,36,39,40] followed the PRISMA guidelines. The publication by Bassi et al. [35] from May 2020 reviewed 50 Indian apps and functions mapped against the guidelines provided by the WHO. Aarogya Setu was found to be the most popular Indian COVID-19 app. The review by Islam et al. [20] included 25 apps from multiple countries, and they visually mapped 26 identified features onto nine objectives. The review by Collado-Borrell et al. [36] enumerated the characteristics of 114 identified apps that were available in August 2020. They discussed how, contrary to other reviews of health-related apps, many of the reviewed COVID-19 apps have been designed by governments. Ming et al. [37] analyzed 58 apps for self-monitoring and education. In total, 58.3% (*n* = 28) of the apps scored at least four points on a seven-point scale proposed by Nouri et al. [54]. In feature assessments, apps from Apple and Android scored a mean of three and two points, respectively, on a five-point scale by Izahar et al. [55]. Finally, they provided recommendations for apps for COVID-19. Alanzi [18] provided an overview of 12 COVID-19 apps from Saudi Arabia, Italy, Singapore, the UK, and the USA. In total, 75% (*n* = 9) of the apps were contact-tracing apps (CTAs). He discussed the potential benefits of an integrated application that contained multiple features as features were at the time spread across multiple apps. By using the open-coding technique, Almalki and Gianncicci [21] identified 29 key technical features in 115 apps, out of which they created a taxonomy that included five COVID-19 app purposes. The two most frequent technical features were basic health information (36.52%; *n* = 42) and contact tracing (27.83%; *n* = 32). Zhang et al. [38] identified 103 commercial COVID-19 apps and showed a steady increase in app publications from February to April 2020. The review by Lee et al. [39] identified 46 free COVID-19 apps by governments from 11 countries within East and Southeast Asia alongside key characteristics and functions. Most apps (70%; *n* = 32) were intended for the general public, the most used technology was GPS (61%; *n* = 28), and usage was mandatory for 52% of the applications (*n* = 24). Erfannia et al. [40] evaluated four Persian apps with a self-made checklist consisting of 37 yes or no questions. All apps performed well regarding ease of use and privacy while needing improvement in education, monitoring, and data sharing.

#### 3.3.2. Privacy and Security

Six reviews [19,41,42,43,44,45] were grouped as they all assessed apps for COVID-19 concerning either privacy or security. Singh et al. [19] presented a review of 29 apps and how apps from 19 countries differed in their degree of privacy invasion. They showed how CTAs supported real-time location tracking, including data from public surveillance systems, government information systems, or credit card transactions, while other countries used GPS-based geofencing technology to enforce the quarantine of individuals. Hatamian et al. [41] analyzed 28 Android-based CTAs regarding their privileges, privacy policies, run-time permission access, and vulnerabilities. CTAs generally required more permissions than needed and only partly justified their request. In total, 64.3% (*n* = 18) of apps did not fulfill half of the 12 policy principles proposed by Hatamian et al. [56]. No policy enforced noticing users upon a privacy breach as required by the European General Data Protection Regulation (GDPR). In total, 61% (*n* = 17) of the apps requested at least one form of location interface. Apps from the EU generally requested fewer and less privacy-invasive permissions, had higher-quality policies, and were more secure. Nazayer et al. [42] discussed how centralized app architectures provide more data to track secondary infections and perform research, while decentralized architectures provide a higher level of privacy. They further argue how a collection of more data can increase functionality at the cost of user privacy and how the integration of multiple technologies in one COVID-19 CTA could increase overall benefits. Within the review by Kouliaridis et al. [43], static code analysis exposed apps that were potentially susceptible to common weakness enumerations (CWE; 62%), as well as issues with apps’ manifest files (88%), shared libraries (46%), outdated software components (25%), or data leakage (33%). Kolasa et al. [44] developed two checklists based on a report by the Ada Lovelace Institute [57], the privacy code of conduct for mobile health apps from the European Commission [58], and the guidelines on the use of location and contract-tracing tools in the context COVID-19 from the European Data Protection Board [59], through which they found differing balances between data privacy and public health interests, which they attributed to socio-geographical differences. The systematic literature review of 40 studies by Alshawi et al. [45] found that while Asian countries often trade in privacy in the name realm of public health via mandatory app uptake, other countries’ app adoption rates struggle with civic acceptance. They then demonstrate how governments around the world vary greatly in privacy protection and point out the need for policies that ensure such protection.

#### 3.3.3. App Reviews Using the Mobile Application Rating Scale

**Figure 5:**
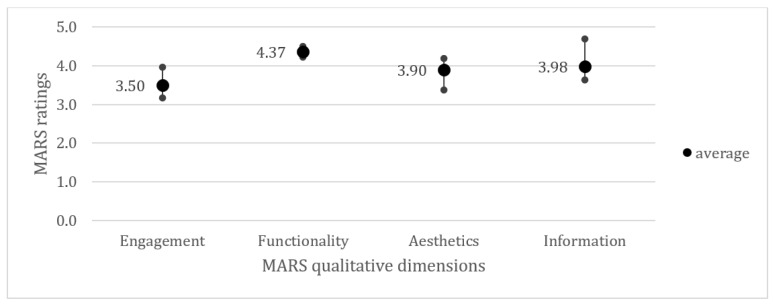
An overview of the average (with minimum and maximum) ratings of MARS dimensions across all five reviews.

Five reviews [32,47,48,49,50,60] rated COVID-19 apps using MARS. Across all five reviews, the overall mean MARS scores of all analyzed apps were above the possible mean (3.7 [60], 4.2 [47], 4.07 [48], 3.97 [49], 3.81, and 3.56 [50]). Except for Salehinejad et al. [47], all reviews rated the “Functionality” dimension as the best and “Engagement” as the worst on average, as Figure 5 demonstrates.

Davalbhakta et al. [60] provided examples of good design choices concerning each MARS dimension. They generalized that apps from India usually scored higher in functionality, while apps from the UK and the USA scored higher in information dissemination. While assessing national and international apps for COVID-19, Salehinejad et al. [47] pointed out the focus of developers on functionality, identifying the engagement and aesthetics dimensions as potential target areas for improvements. In their rating of the 16 most popular mental health apps according to Carlo et al. [61], Wang et al. [48] found an increase in interest in mental health apps, which they attributed in part to an increase in mental health issues during the pandemic. The review of Kahnbach et al. [49] investigated the quality characteristics of 21 national European COVID-19 CTAs using the German modification of MARS (MARS-G) by Messner et al. [62]. They found a positive correlation between app quality and app adoption rate. Acknowledging that the Chinese government has spread pandemic apps across several marketplaces, Fan et al. included 20 apps that were either independent apps or WeChat applets. They noted a diversity of regional apps, which reduced their usability as users traveled. They also discussed the impact of the mandatory use of certain COVID-19 apps and QR codes issued by the Chinese government.

#### 3.3.4. Miscellaneous

Four reviews [22,51,52,53] were grouped as “Miscellaneous”. In their assessment using the tool by the Effective Public Health Practice Project (EPHPP) [63], Kondylakis et al. [22] found moderate quality for two (17%) and weak methodological quality for ten (83%) studies. They suspected that the authors desired to publish quickly at the beginning of the pandemic and lastly summarized implications for clinical practice. Akinbi et al. [51] inspected challenges and future directions for CTAs in neo-liberal societies via a systematic literature review; privacy concerns were the most popular subject (46%). They discussed the importance of adopting privacy-preserving technologies and maintaining a high level of transparency, a human-centered development of CTAs, and ethical considerations that prevent the disadvantage of parts of the population. Blacklow et al. [52] presented a 14-item evaluation framework through which they analyzed 26 apps from the USA with a focus on accessibility and inclusivity. In total, 69% of the apps exceeded 9th-grade readability in the context of a referenced average reading level of 7th–8th grade in the U.S. [64]. Moreover, 65% of the apps were available only in English, and 69% of the apps did not include videos or illustrations to explain how they function. Reviewing the literature on the effectiveness of CTAs on epidemiological outcomes, Jenniskens et al. [53] judged two observational–comparative studies to be of low methodological quality. The other 15 model-based studies indicated the benefits of CTAs on the reproduction rate (R), as well as the rates of infection and mortality.

### 3.4. Quality Assessment

The AMSTAR ratings of each of the included reviews are shown in Table 3. The detailed rating by item can be found in the Supplementary Materials S6. The quality rating was low, with most studies having a rating of “low” (*n* = 14), followed by “critically low” (*n* = 8) and “moderate” (*n* = 2).

## 4. Discussion

### 4.1. Principal Results

We present a high-level overview of 24 reviews on mobile apps for COVID-19 that were published between January 1st 2020 and April 25th 2022. Most reviews were published before July 2021. Out of the 24 reviews, the majority were reviews of apps (71%), followed by reviews of the published literature (21%) and hybrid reviews that looked at both apps and the literature (8%). The overall quality of the included reviews is low with respect to the majority of articles. Most articles either achieved a “critically low” (*n* = 8) or “low” (*n* = 14) AMSTAR rating. Only two articles received a “moderate” rating. Little research has been performed on apps from Africa and Central and South America. Overview reviews identified that many apps that have been published globally were mostly developed under supervision from national or local governments and used common technologies, such as Bluetooth and GPS, to perform their purposes. Reviews with a focus on privacy and security reported differing degrees of privacy invasion across countries and security vulnerabilities within apps. Generally, Asian countries collected more user data than in Europe or North America, and usage was more often mandatory. Reviews that rated apps by MARS consistently found high-quality apps and identified the most potential regarding further improvement in making apps more engaging. Other reviews identified privacy concerns to be the main factor keeping people in neo-liberal societies from using CTAs, reported bad accessibility and inclusivity in apps, and found a lack of methodologically sound studies that evaluated mobile apps for COVID-19.

Considering that the WHO declared SARS-CoV-2 a pandemic in March 2020 [3], most reviews from 2020 were published rather quickly (Figure 2). The fast publication time could be attributable to a global focus on the COVID-19 pandemic and scholars’ desire to share results as quickly as possible. The visible gap after July 2021 may reflect a lower sense of urgency around COVID-19, driven by less lethal virus variants such as the omicron strand [65], steadily increasing vaccination rates [66], and more accurate information about how to manage the risks of COVID-19. There was a large geographical bias in the research on COVID-19 mobile apps. Many articles focused on apps from the USA, Europe, and India, for which, aside from their strong academic sectors, one could assume a correlation in case numbers to be the reason. African and Central and South American countries have mostly been ignored so far, emphasizing the need for research on apps developed by them.

COVID-19 mobile apps have served purposes from simple ones such as information dissemination and symptom monitoring to complex ones like contact tracing. With more development over time, apps have been extended to support additional features that reflect the introduction of COVID-19 vaccinations and changing legal contexts. The share of apps with a governmental background is untypically high for health apps [36]. This is likely due to restrictions that Google [67] and Apple [68] have put in place for COVID-19 apps to ensure the credibility of apps and the information shared. Governments in many Asian countries are able to implement more privacy-invasive features, as there are fewer legal restrictions compared to European or North American countries, and mandatory app uptake ensured that a sufficient number of people used the apps [19,35,39,41,42,44,45]. In particular, in European countries, the efficiency of certain features was limited by the strict data privacy regulation through the GDPR and voluntary app uptake. As Akinbi et al. concluded, people in neo-liberal societies, especially within Europe, were naturally skeptical towards their governments and tended to question privacy invasion [51]. Alshawi, via an example of France, demonstrated how people may refuse to use CTAs due to this skepticism, which logically would decrease their effectiveness [45,69]. The reported security vulnerabilities in popular CTAs [41,43] are especially critical as health data are one of the most vulnerable types of data and also because public acceptance is built on trust that as little data as possible are collected and that these data are safe from abuse and theft. The overall very high total ratings across MARS reviews [47,48,49,50,60] likely reflect the global focus and the governmental support in developing them. Although some authors referred to “above-average” ratings (Refs. [49,60]), as we are unaware of a published average MARS score, such a benchmark still needs to be developed. In the context of MARS, making apps more engaging seemed to have the greatest potential in increasing app quality [48,49,50,60], with better app quality contributing to increased app adoption [49]. The gap in accessibility and inclusivity regarding required reading levels, available languages, and the extent to which app functions were described by appropriate illustrations that were pointed out by Blacklow et al. [52] reveals another dimension for improvement. In particular, in the context of medical knowledge that may seem complex and even intimidating to people without a medical background, together with a continuously globalized world, having several supported languages that are appropriate to the geographical context and easily understandable is critical for public acceptance and, consequently, app uptake. While reviews of evaluation studies indicated a positive influence of CTAs on pandemic progression, they also pointed out a lack of evidence due to methodological weaknesses [22,53]. In this context, as retrospective studies struggle with many confounders, methodologically rigorous study designs need to be developed now to prepare for possible future epidemic situations.

Wangler and Jansky [70] discuss concerns concerning the clarity of the content, transparency, and privacy in mHealth applications. In a study, a significant amount of reviewed mHealth applications had poor quality and did not follow best practices in data security [71]. Patients often do not use the mHealth application that they have installed because of security concerns, loss of interest, costs, or badly designed user interfaces [72].

### 4.2. Limitations

As the review protocol did not meet the inclusion criteria of the protocol publishing platform PROSPERO, it could not be published before the review. This reduces transparency and makes the review susceptible to bias because we cannot prove that we did not deviate from the original protocol.

Although screening was carried out by two reviewers operating independently, all qualitative reviews may be influenced by reviewers’ subjectivity. It is therefore possible that other scholars could obtain different results when using the same dataset.

This review is limited by the timeframe of the database search and our narrow search strategy. Records published or updated after our search or research that did not meet our inclusion criteria were not considered [73]. It is possible that we thus unintentionally failed to include some relevant research. We only searched two databases; the search of additional databases would have led to more results. The fact that we only included reviews of COVID-19-related apps is a limitation, as other research that, for example, studied factors contributing to the success and failure of contact-tracing systems [74] were not included in this review of reviews and could have provided additional insights.

### 4.3. Comparison with Prior Work

To our knowledge, this is the first systematic review of reviews (umbrella review) on the topic of mobile apps for COVID-19. A number of reviews have been published that were included in our work, but a review of reviews that summarizes the findings of these reviews has not been published so far. In addition to providing this high-level overview of the state of the literature about apps for COVID-19, the results of our review can serve as a unique data source for future research via secondary data analysis to address more specific research objectives about this topic.

## 5. Conclusions

We present a high-level overview of 24 reviews on mobile apps for COVID-19 during the first two and a half years of the pandemic. There appeared to be a lower sense of urgency to publish research on mobile apps for COVID-19 after July 2021. The quality of the included reviews is quite low. Further, we observe a need for research on COVID-19 mobile apps from African and Central and South American countries, as apps from these countries are currently ignored in the literature. Apps were mostly developed with governmental backgrounds, which was reflected by consistent reports of high app quality according to MARS. Although reviews that evaluated studies on COVID-19 mobile apps regarding their effectiveness indicated positive influences of these apps on pandemic progression, there is yet no evidence due to methodological deficits. Future research is needed on means to maximize voluntary app uptake in possible future epidemics, including data minimization, transparency, and user engagement. The development of rigorous and methodological study designs can prepare the generation of evidence regarding the use of future mobile apps for epidemics.

## Figures and Tables

**Table 1:** Key eligibility features are identified using the PICO framework.

Dimension	Description
Population	People at risk of being exposed to SARS-CoV-2 or who were diagnosed with COVID-19
Intervention	Reviews of COVID-19-related apps
Control	With or without a comparator
Outcome	Metadata Review’s subject Methods used Results

**Table 2:** Search strings for the respective databases.

Database	Search String
Medline	*(“SARS-CoV-2”[Mesh] OR SARS-CoV-2[tw] OR COVID-19[tw] OR CORONA) AND (“Mobile Applications”[Mesh] OR Mobile Applications[tw] OR Smartphone[tw] OR Telemedicine [tw])*
Embase	*(‘mobile application’/exp OR ‘mobile application’ OR ‘smartphone’/exp OR smartphone OR ‘mobile phone’/exp OR ‘mobile phone’ OR ‘telemedicine’/exp OR ‘telemedicine’) AND (‘coronavirus disease 2019’ OR ‘19’)*

**Table 3:** The 24 included reviews sorted by category with the AMSTAR rating.

Study ID	Title	Objective	Category	AMSTAR Rating
Bassi 2020 [35]	An overview of mobile applications (apps) to support the coronavirus disease 2019 response in India	To identify COVID-19-related mobile apps and highlight gaps to inform the development of future mHealth initiatives.	Overview	Low
Islam 2020 [20]	A Review on the Mobile Applications Developed for COVID-19: An Exploratory Analysis	To explore the existing mobile applications developed for the COVID-19 pandemic.	Overview	Low
Collado-Borrell 2020 [36]	Features and Functionalities of Smartphone Apps Related to COVID-19: Systematic Search in App Stores and Content Analysis	To identify smartphone apps designed to address the COVID-19 pandemic and analyze their characteristics.	Overview	Low
Ming 2020 [37]	Mobile Health Apps on COVID-19 Launched in the Early Days of the Pandemic: Content Analysis and Review	To analyze and evaluate the contents and features of COVID-19 mobile apps.	Overview	Critically low
Alanzi 2021 [18]	A Review of Mobile Applications Available in the App and Google Play Stores Used During the COVID-19 Outbreak	To review the functionalities and effectiveness of mHealth apps during the COVID-19 outbreak.	Overview	Critically low
Almalki 2021 [21]	Health Apps for Combating COVID-19: Descriptive Review and Taxonomy	To categorize health apps related to COVID-19, explore their key technical features, and classify their purposes.	Overview	Low
Zhang 2021 [38]	An Overview of Commercially Available Apps in the Initial Months of the COVID-19 Pandemic	To identify the commercial applications that are currently available for COVID-19 and explore their functionalities.	Overview	Critically low
Lee 2021 [39]	Mobile Apps Leveraged in the COVID-19 Pandemic in East and South-East Asia: Review and Content Analysis	To examine free apps from East and Southeast Asian countries, highlight their key characteristics, and interpret the relation of apps’ release dates and commencement dates of other COVID-19 public health policies.	Overview	Low
Erfannia 2022 [40]	Reviewing and Content Analysis of Persian Language Mobile Health Apps for COVID-19 Management.	To carry out a content analysis of free Persian mobile health apps in the management of COVID-19 and determine the relationship between the popularity and quality of these apps.	Overview	Low
JohnLeonSingh 2020 [19]	Mobile Health Apps That Help With COVID-19 Management: Scoping Review	To scope the evidence base on apps that were developed in response to COVID-19.	Privacy and Security	Low
Hatamian 2021 [41]	A privacy and security analysis of early-deployed COVID-19 contact tracing Android apps.	To analyze the privacy and security performance of Android contact-tracing applications, including code privileges, promises, privacy policies, and static and dynamic performance.	Privacy and Security	Critically low
Nazayer 2021 [42]	Contact-tracing applications: A review of technologies	To examine design decisions related to COVID-19 contact-tracing applications and the implications of these decisions.	Privacy and Security	Critically low
Kouliaridis 2021 [43]	Dissecting contact tracing apps in the Android platform.	To analyze all the official Android contact-tracing apps deployed by European countries regarding privacy and security via static and dynamic code analysis.	Privacy and Security	Critically low
Kolasa 2021 [44]	State of the Art in Adoption of Contact Tracing Apps and Recommendations Regarding Privacy Protection and Public Health: Systematic Review	To analyze available COVID-19 contact-tracing apps and verify to what extent public health interests and data privacy standards can be fulfilled simultaneously in the process of the adoption of digital health technologies.	Privacy and Security	Low
Alshawi 2022 [45]	Data privacy during pandemics: a systematic literature review of COVID-19 smartphone applications.	To provide a better study of privacy concerns in the context of COVID-19 apps, examine and analyze existing studies on COVID-19 apps and privacy concerns and their findings, and provide summaries.	Privacy and Security	Critically low
Davalbhakta 2020 [46]	A Systematic Review of Smartphone Applications Available for Corona Virus Disease 2019 (COVID19) and the Assessment of their Quality Using the Mobile Application Rating Scale (MARS)	To assess mobile applications for COVID-19 using the Mobile Application Rating Scale.	MARS	Moderate
Salehinejad 2021 [47]	A review and content analysis of national apps for COVID-19 management using Mobile Application Rating Scale (MARS)	To develop a reliable measure and rate the quality of COVID-19 mobile health apps.	MARS	Low
Wang 2021 [48]	Investigating Popular Mental Health Mobile Application Downloads and Activity During the COVID-19 Pandemic.	To analyze downloads and the user activity of select popular mental health apps during COVID-19	MARS	Low
Kahnbach 2021 [49]	Quality and Adoption of COVID-19 Tracing Apps and Recommendations for Development: Systematic Interdisciplinary Review of European Apps	To investigate the quality characteristics of national European COVID-19 contact-tracing apps, investigate associations between app quality and adoption, and identify app features contributing to higher app quality.	MARS	Low
Fan 2022 [50]	The function and quality of individual epidemic prevention and control apps during the COVID-19 pandemic: A systematic review of Chinese apps.	To investigate the functional characteristics of individual epidemic prevention and control apps in China and evaluate their quality.	MARS	Moderate
Kondylakis 2020 [22]	COVID-19 Mobile Apps: A Systematic Review of the Literature	To review studies that have used and evaluated mobile apps for COVID-19.	Miscellaneous	Low
Akinbi 2021 [51]	Contact tracing apps for the COVID-19 pandemic: a systematic literature review of challenges and future directions for neo-liberal societies	To encompass current challenges facing contact-tracing applications and recommendations that address such challenges in the fight against the COVID-19 pandemic in neo-liberal societies.	Miscellaneous	Critically low
Blacklow 2021 [52]	Usability, inclusivity, and content evaluation of COVID-19 contact tracing apps in the United States.	To evaluate COVID-19 contact-tracing apps via an evaluation framework with objective measures of usability that are presented in this work.	Miscellaneous	Low
Jenniskens 2021 [53]	Effectiveness of contact tracing apps for SARS-CoV-2: A rapid systematic review	To systematically review evidence on the effectiveness of contact-tracing apps (CTAs) for SARSCoV-2 on epidemiological and clinical outcomes.	Miscellaneous	Low

## Data Availability

The datasets used and analyzed during the current study are available from the corresponding author upon request.

## Supplementary Materials

The following supporting information can be downloaded at https://www.mdpi.com/article/10.3390/healthcare12020139/s1. Table S1: Review protocol; Table S2: All included studies; Table S3: Records excluded during full-text-screening together with reasons for exclusion; Table S4: Data extraction template; Table S5: AMSTAR template; Table S6: Quality rating using the modified AMSTAR.
