# Supplementary material for: Mobile Apps for COVID-19: A Systematic Review of Reviews
Source: Healthcare (Basel). 2024 Jan 8;12(2):139. doi: 10.3390/healthcare12020139 (PMC10815093; doi:10.3390/healthcare12020139)
Supplement: Supplementary file 1 [file healthcare-12-00139-s001.zip › S1.pdf]

Review Protocol:
Mobile Apps for COVID 19: A Systematic Review of Reviews

Administrative Information

Section and topic	Item No	Checklist Item		
**Title:**				
**Identification**	1a	Protocol for a quasi-systematic review of reviews on mobile apps for Covid-19		
**Update**	1b	n/a (This protocol is not for an update for a previous systematic review)		
**Registration**	2	n/a (This protocol is not yet registered)		
**Authors:**				
**Contact**	3a	Felix Holl, M.Sc.	Research associate, DigiHealth Institute, Neu-Ulm	Felix.Holl@hnu.de
		Marco Aigner, B.Sc.	Graduate assistant, DigiHealth Institute, Neu-Ulm	Marco.Aigner@student.hnu.de
**Contributions**	3b	0. Conceptualization: Felix Holl & Marco Aigner<br>1. Database search: Marco Aigner<br>2. Title/Abstract & Full text Screening: Felix Holl & Marco Aigner<br>3. Data Analysis: Felix Holl & Marco Aigner<br>4. Manuscript Writing: Felix Holl & Marco Aigner<br>5. Manuscript Review and Editing: Felix Holl, Marco Aigner, Johannes Schobel & Walter Swoboda		
**Amendments**	4	Important protocol amendments shall be documented		
**Support**	5	No funding to declare		

Introduction

Section and topic	Item No	Checklist Item
**Rationale**	6	• To identify and assess already existing reviews on apps for Covid-19<br>• To find out whether further research of current health apps for Covid-19 is necessary.
**Objectives**	7	• What previous work has been done to assess current native health apps for Covid-19 (in the United States)?<br>• PICO<br>○ Patients: People at risk of being exposed to SARS-CoV-2 or being diagnosed with Covid-19<br>○ Intervention: Reviews of mobile apps for Covid-19<br>○ Control: With or without a comparator<br>○ Outcome: Metadata, subject of review, methods used, results

Methods

Section and topic	Item No	Checklist Item	
**Eligibility criteria**	8	PICO	See Item No 7
		Study design	Reviews
		Setting	See Item No 7
		Time frame	n/a
		Years considered	01/01/2020 - today
		Language(s)	English
		Publication status	Peer-Reviewed & Published
**Information Sources**	9	• Databases:<br>○ Medline<br>○ Embase<br>• No other sources planned	
**Search strategy**	10	1. Initial Research	

		a. Search databases (see item no 9)<br>b. Eligibility criteria (see item no 8)<br>2. Title/Abstract Screening<br>a. Screening by two independent researchers<br>b. Discussion on conflicts and agreement on in-/ or exclusion<br>3. Paper retrieval<br>a. Exclude papers that could not be retrieved<br>4. Full-text screening by two independent researchers<br>a. Discussion on conflicts and agreement on in-/ or exclusion<br>i. Documentation of reasons for exclusion<br>5. Analysis and synthesis of remaining studies<br>a. Risk of bias
**Study records:**		
**Data management**	11a	1. Storage of studies<br>○ Result sets are being exported from the databases using text-, .csv or proprietary file-formats<br>○ Results are being imported into Endnote 20<br>2. The review process (selection, discussion on in-/ or exclusion) is carried out in Covidence
**Selection process**	11b	See item no 10
**Data collection process**	11c	n/a
**Data items**	12	• Reviews<br>○ Metadata<br>○ Subject of review<br>○ Methods Used<br>○ Results<br>• Apps<br>○ Total

		○ Categories<br>○ Countries<br>○ Assessment/Ratings
**Outcomes and prioritization**	13	• Metadata of studies<br>• Categorization of studies<br>• Study results
**Risk of bias in individual studies**	14	• Bias in qualitative studies is to be assesses using the GRADE-CERQual approach.
**Data synthesis**	15a	• Criteria under which study data will be quantitatively synthesized<br>○ Number of assessed apps<br>○ Categories of assessed apps<br>○ Methods used to assess apps<br>○ Results of app assessments
	15b	• Depending on the methods found in studies
	15c	• No additional analyses (sensitivity or subgroup analyses, meta-regression) are planned
	15d	• Qualitative synthesis with regard to possible effects of mobile apps on Covid-19 or the need to perform further research on the topic
**Meta-bias(es)**	16	n/a
**Confidence in cumulative evidence**	17	n/a
