# Supplementary material for: Mobile Apps for COVID-19: A Systematic Review of Reviews
Source: Healthcare (Basel). 2024 Jan 8;12(2):139. doi: 10.3390/healthcare12020139 (PMC10815093; doi:10.3390/healthcare12020139)
Supplement: Supplementary file 1 [file healthcare-12-00139-s001.zip › S3.pdf]

Table 1 Records excluded during full-text-screening together with reasons for exclusion

**Study**	**Reason for exclusion**
Thomas 2021	Wrong intervention
Guisado-Clavero 2021	Wrong intervention
Gabarron 2021	Wrong intervention
Abd-Alrazaq 2021	Wrong intervention
Scott 2020	Wrong intervention
Storeng 2021	Wrong intervention
Abbaspur-Behbahani 2022	Wrong intervention
Asadzadeh 2021	Wrong intervention
Alonso 2021	Wrong intervention
Osmanlliu 2021	Wrong intervention
Eslami 2021	Wrong intervention
Silenou 2021	Wrong intervention
Grekousis 2021	Wrong intervention
Lukas 2020	Wrong intervention
Alo 2021	Wrong intervention
Aslani 2020	Wrong intervention
Iyengar 2020	Wrong intervention
Kumar 2020	Wrong intervention
Mbunge 2020	Wrong intervention
LaiSHS 2021	Wrong intervention
Leite 2021	Wrong intervention
Mehraeen 2022	Wrong intervention
García-Iglesias 2020	Wrong Language
Geissbuhler 2020	Wrong Language
Wongsin 2022	Wrong outcomes
Wirth 2020	Wrong setting
Elkhodr 2021	Wrong type of papeer
Verhagen 2020	Wrong type of papeer
Du 2020	Wrong type of papeer
Wang 2021	Wrong type of papeer
Weiß 2021	Wrong type of papeer
Dixon 2021	Wrong type of papeer
Vandamme 2020	Wrong type of papeer
Hogan 2021	Wrong type of papeer
Grantz 2020	Wrong type of papeer
Chaudhary 2021	Wrong type of papeer
Colizza 2021	Wrong type of papeer
No Author 2020	Wrong type of papeer
Jahnel 2020	Wrong type of papeer

Sharma 2020	Wrong type of papeer
Toussaert 2021	Wrong type of papeer
Wu 2021	Wrong type of papeer
Hernandez-Orallo 2020	Wrong type of papeer
Khan 2021	Wrong type of papeer
Wang 2021	Wrong type of papeer
Min-Allah 2021	Wrong type of papeer
