# Supplementary material for: Mobile Apps for COVID-19: A Systematic Review of Reviews
Source: Healthcare (Basel). 2024 Jan 8;12(2):139. doi: 10.3390/healthcare12020139 (PMC10815093; doi:10.3390/healthcare12020139)
Supplement: Supplementary file 1 [file healthcare-12-00139-s001.zip › S4.pdf]

Data extraction template

Item	Format/Description	Options
Study ID	ID assigned by Covidence (after the #)	
Title		
Lead author contact details		
Journal	Published in	
Publication Date	DD.MM.YYYYY	
Type of study		App-Review<br>Literature Review<br>App and Literature Review<br>Other
Research Question		
Notes	Room for optional additional notes	
Methods	Heading 1	
App Search	Heading 2	
App search?	Were apps searched	Yes<br>No
App search start	DD.MM.YYYY	
App search end	DD.MM.YYYY	
App search time	If the exact time span is not given, use this field instead of "app search start" and "app search end"	
Search platforms		Google Play Store<br>Apple App Store<br>Other
App search strings	Separated by commas	
App search method		
App search steps		
App countries	Countries from which apps were sought.<br>Separated by commas	
App inclusion	Separated by commas	
App exclusion	Separated by commas	

App Analyzed variables	Separated by commas	
App rating		Yes<br>No
App rating method	e.g. MARS, ...	
App categorization	Have the apps been categorized by the reviewers?	Yes<br>No
App categorization method		
Literature Search	Heading 2	
Literature Search	Was literature searched and reviewed within the paper?	Yes<br>No
Using PRISMA	Does the study use any variant of the PRISMA approach for systematic reviews?	Yes<br>No
PRISMA guideline	If available, which version of PRISMA has been used?	
Literature search start	DD.MM.YYYY	
Literature search end	DD.MM.YYYY	
Literature search time	If the exact time span is not given, use this field instead of "literature search start" and "literature search end"	
Literature databases	Which databases were used for literature review? Separated by commas	
Literature search strings	Separated by commas	
Literature inclusion	Separated by commas	
Literature exclusion	Separated by commas	
Literature search steps		
Literature analyzed variables		
Results	Heading 1	
App search	Heading 2	
Total apps	Total of apps included in the analysis.	
App findings		
Literature search	Heading 2	

Total studies		
Literature findings		
Discussion	Heading 1	
App discussion	Discussion related to app review	
Literature discussion	Discussion related to literature review	
Other	Heading 1	
Limitations		
Future studies	Recommendations for future studies	
Study funding sources		
Possible conflicts of interest for the authors		
