# Supplementary material for: Mobile Apps for COVID-19: A Systematic Review of Reviews
Source: Healthcare (Basel). 2024 Jan 8;12(2):139. doi: 10.3390/healthcare12020139 (PMC10815093; doi:10.3390/healthcare12020139)
Supplement: Supplementary file 1 [file healthcare-12-00139-s001.zip › S5_AMSTAR-Template.pdf]

Template for Quality rating using the modified AMSTAR

AMSTAR 1. Inclusion of PICO?
Did the research questions and inclusion criteria for the review include the components of PICO?

Population, Intervention, Comparator group, Outcome, Timeframe to follow up
1. Yes
2. No

Extractors will also be able to add supporting text to justify their judgements

AMSTAR 2. Adherence to protocol?*
Did the report of the review contain an explicit statement that the review methods were established prior to the conduct of the review and did the report justify any significant deviations from the protocol?

Can mean inclusion of review questions, search strategy, inclusion/exclusion, risk of bias assessment,
1. Yes
2. Partial Yes
3. No

Extractors will also be able to add supporting text to justify their judgements

AMSTAR 4. Comprehensive search strategy?*
Did the review authors use a comprehensive literature search strategy?

At least 2 databases, keywords and search strategy provided, publication restrictions justified
1. Yes
2. Partial Yes
3. No

Extractors will also be able to add supporting text to justify their judgements

AMSTAR 5. Duplicate study selection
Did the review authors perform study selection in duplicate?

At least two reviewers independently agreed on selection and achieved consensus OR two reviewers selected a sample of eligible studies and achieved good agreement (>= 80 %)
1. Yes
2. No

Extractors will also be able to add supporting text to justify their judgements

AMSTAR 6. Duplicate data extraction
Did the review authors perform data extraction in duplicate?

At least two reviewers achieved consensus on which data to extract OR two reviewers extracted data from a sample of eligible studies and achieved good agreement (>= 80 %)

1. Yes
2. No

Extractors will also be able to add supporting text to justify their judgements

AMSTAR 7. Justification of exclusion*

Did the review authors provide a list of excluded studies and justify the exclusions?

1. Yes
2. Partial Yes
3. No

Extractors will also be able to add supporting text to justify their judgements

AMSTAR 8. Detailed study description

Did the review authors describe the included studies in adequate detail?

Population, Intervention, Comparators, Outcomes, Research Designs

1. Yes
2. Partial Yes
3. No

Extractors will also be able to add supporting text to justify their judgements

AMSTAR 10. Reporting of funding

Did the review authors report on the sources of funding for the studies included in the review?

1. Yes
2. No

Extractors will also be able to add supporting text to justify their judgements

AMSTAR 16. Conflicts of interest?

Did the review authors report any potential sources of conflict of interest, including any funding they received for conducting the review?

1. Yes
2. No

Extractors will also be able to add supporting text to justify their judgements
