# Supplementary material for: Mobile Apps for COVID-19: A Systematic Review of Reviews
Source: Healthcare (Basel). 2024 Jan 8;12(2):139. doi: 10.3390/healthcare12020139 (PMC10815093; doi:10.3390/healthcare12020139)
Supplement: Supplementary file 1 [file healthcare-12-00139-s001.zip › S6_Quality rating using the modified AMSTAR.pdf]

*Quality rating using the modified AMSTAR with critical items marked with an **

Item number	1	2*	4*	5	6	7*	8	10	16	AMSTAR Rating
Alanzi 2020	No	No	Yes	No	No	No	Yes	No	Yes	Critically low
Almalki 2021	No	No	Yes	No	Yes	Partial Yes	Yes	Yes	Yes	Low
Bassi 2020	No	No	Yes	No	No	Partial Yes	Yes	Yes	Yes	Low
Blacklow 2021	No	No	Yes	No	Yes	Partial Yes	Yes	Yes	Yes	Low
Collado-Borrell 2020	No	No	Yes	No	Yes	No	Partial Yes	Yes	Yes	Low
Davalbhakta 2020	No	Yes	Yes	Yes	Yes	Yes	Yes	No	Yes	Moderate
Erfannia 2022	No	No	Partial Yes	No	No	Yes	No	No	No	Low
Fan 2022	No	Partial Yes	Yes	No	No	Partial Yes	Yes	Yes	Yes	Moderate
Islam 2020	No	No	Yes	Yes	Yes	Partial Yes	Yes	No	No	Low
JohnLeonSingh 2020	No	No	Yes	No	No	Partial Yes	Yes	No	Yes	Low
Kahnbach 2021	No	No	Yes	No	Yes	Yes	Yes	No	Yes	Low
Kondylakis 2020	No	No	Yes	Yes	No	Partial Yes	Yes	Yes	Yes	Low
Kouliaridis 2021	No	No	No	No	Yes	No	Yes	No	No	Critically Low
Lee 2021	No	No	Yes	Yes	Yes	Yes	Partial Yes	No	Yes	Low
Ming 2020	No	No	Yes	No	No	No	Yes	No	Yes	Critically low
Nazayer 2021	No	No	No	No	No	Partial Yes	Partial Yes	Yes	Yes	Critically low
Salehinejad 2021	No	No	Yes	No	No	Partial Yes	Yes	Yes	Yes	Low
Wang 2021	No	No	Yes	No	No	Partial Yes	Yes	No	No	Low
Akinbi 2021	No	No	Yes	No	No	No	Yes	Yes	Yes	Critically low
Hatamian 2021	No	No	Yes	No	Yes	No	Yes	Yes	No	Critically low
Jenniskens 2021	No	No	Yes	Yes	Yes	Yes	Yes	Yes	Yes	Low
Kolasa 2021	No	No	Yes	Yes	No	Yes	Yes	Yes	Yes	Low
Zhang 2021	No	No	Partial Yes	Yes	No	No	No	Yes	No	Critically low
Alshawi 2022	No	No	Yes	No	No	No	Yes	Yes	Yes	Critically low
